# Targeting CD8 T-Cell Metabolism in Transplantation

**DOI:** 10.3389/fimmu.2015.00547

**Published:** 2015-10-23

**Authors:** Michelle Yap, Sophie Brouard, Claire Pecqueur, Nicolas Degauque

**Affiliations:** ^1^UMR 1064, INSERM, Nantes, France; ^2^Faculté de Médecine, Université de Nantes, Nantes, France; ^3^CHU de Nantes, ITUN, Nantes, France; ^4^CIC Biothérapie, Nantes, France; ^5^CHU Nantes, CRB, Nantes, France; ^6^UMR 892, INSERM, Nantes, France

**Keywords:** CD8 lymphocytes, transplantation, metabolism, glycolysis, mitochondria, regulation, therapeutics

## Abstract

Infiltration of effector CD8 T cells plays a major role in allograft rejection, and increases in memory and terminally differentiated effector memory CD8 T cells are associated with long-term allograft dysfunction. Alternatively, CD8 regulatory T cells suppress the inflammatory responses of effector lymphocytes and induce allograft tolerance in animal models. Recently, there has been a renewed interest in the field of immunometabolics and its important role in CD8 function and differentiation. The purpose of this review is to highlight the key metabolic pathways involved in CD8 T cells and to discuss how manipulating these metabolic pathways could lead to new immunosuppressive strategies for the transplantation field.

## Introduction

To efficiently protect mammalian against pathogen infection, the response of the adaptive T-cell immune system follows a three-step process: an initial phase of T-cell priming followed by a massive clonal expansion and differentiation, a contraction phase leading to the clearance of most effector cells, and the generation of memory immune T cells. Numerous subsets of memory CD8 T cells have been described with different functions and localization within the body. Seminal study by Sallusto et al. identified that isoform CD45RA and lymph node homing marker CCR7 allow the discrimination of naive and subsets of memory CD8 cells (central memory, CM; effector memory, EM; terminally differentiated effector memory cells, TEMRA) (1). The inclusion of additional markers, such as CD27, CD28, CD57, or TBET, can narrow down the identification of specific memory subsets to decipher their relative function during the recall response and to identify checkpoint regulating their differentiation and their survival (2). T-cell response to antigen stimulation and activating their effector functions are energetically demanding processes. Over the last decade, numerous reports have highlighted that the generation of effector and memory cells is not only regulated by the inflammatory milieu but also by metabolic processes.

In the 1920s, Otto Warburg observed that cancer cells consumed much higher amounts of glucose compared with normal cells and that cancer cells mainly utilize glycolysis over mitochondrial respiration, even in the presence of oxygen (3, 4). This metabolic strategy, termed the Warburg effect, has also been observed in activated lymphocytes (5–7). Despite being less efficient albeit rapid at producing ATP, the aerobic glycolysis enables cells to generate the biosynthetic precursors necessary to support rapid growth and proliferation as well as to maintain the redox balance (8). The renewed interest in the metabolic processes involved in T-cell biology has also revealed that evolutionary conserved pathways, such as the mammalian target of rapamycin (mTOR) pathway, are common to both immune responses and metabolism regulation (9). The goal of this review is to summarize the link between CD8 T-cell subsets and their metabolic adaptations and to evaluate how targeting this metabolic programming could be a new possible therapeutic treatment, with a special focus on transplantation.

## T Cell and Transplant Outcome: Known Targets that Required New Therapeutic Tools

Involvement of T lymphocytes in solid organ transplant rejection is a well-researched topic in the transplantation field; however, most of the research focuses on the various roles of the different CD4 lymphocyte subpopulations. Few investigate the role of CD8 cells in transplantation with a main focus on their cytotoxic and proinflammatory functions and how deleterious they are to long-term allograft health (10–13). In acute allograft rejection, infiltrating CD8 T cells play a major role in tubulitis and tissue necrosis. Cytotoxic molecules, such as perforin and granzyme-B, as well as proinflammatory cytokines, such as IFN-γ, secreted by CD8 T cells have been shown to contribute to the rejection response (14, 15). Furthermore, memory CD8 T cells have been shown to act as major barriers for transplant tolerance induction protocols (16, 17). Additionally, recent immunomonitoring research has shown that an increase in certain CD8 populations in transplant patients is associated with allograft rejection and loss. Elevated effector, memory, and TEMRA CD8 subsets in the peripheral circulation are associated with increased occurrence of acute and chronic allograft dysfunction (18–22). As a result, immunomodulation of the CD8 population is an important component for long-term allograft survival and targeting their specific metabolic processes could represent a new mean to foster graft survival.

There is budding interest into a small subpopulation of CD8 lymphocytes with regulatory and suppressive functions. While most of the research has been focused on CD4^+^FOXP3^+^Tregs, there is growing interest in the other types of regulatory T cells, which include the CD8 Tregs (23–25). There have been several different nomenclatures proposed in the literature, which can be used to identify this subset of cells, a few examples being CD8^+^CD28^−^, CD8^+^CD45RC^low^, and CD8^+^FOXP3^+^ (26–28). CD8 Treg populations have potent suppressive ability against effector lymphocytes and promote tolerant induction in rodent transplant models, thus making them an attractive target for promoting allograft survival (23, 24, 28–30). Immunometabolic regulation of CD8 T cells could be used as a means to manipulate the CD8 T-cell immune function for effective immunosuppression. However, little is known regarding their metabolic adaptation during the induction and the expansion phases.

## CD8 T-Cell Metabolic Programming Changes are Specific to CD8 Subsets and Function

### Metabolic Demands in Quiescent T Cells

In resting state, naive T cells rely on oxidative phosphorylation (OXPHOS) to produce adenosine 5′-triphosphate (ATP) (31). This metabolic pathway is the most effective method to generate ATP by catabolizing metabolic intermediates derived from glucose, fatty acids, and amino acids. The low rate of energy metabolism needed by quiescent cells to maintain their housekeeping functions is instructed by cytokine-dependent signals and preferentially IL-7-mediated signals. Indeed, IL-7 is a crucial cytokine to sustain the homeostasis of naive cells and their survival and is also an important regulator of the glucose transporter GLUT-1 (32). The IL-7 receptor (IL-7R) is largely expressed in naive lymphocytes and downregulated when naive CD8 T cells differentiate into the effector phenotype. IL-7 causes activation of JAK1 and JAK3, which in turn activates STAT5 and the PI3K/AKT pathways (32–34). IL-7 allows a basal glucose metabolism, which has been shown to be essential for quiescent T-cell survival (32).

**Figure 1:**
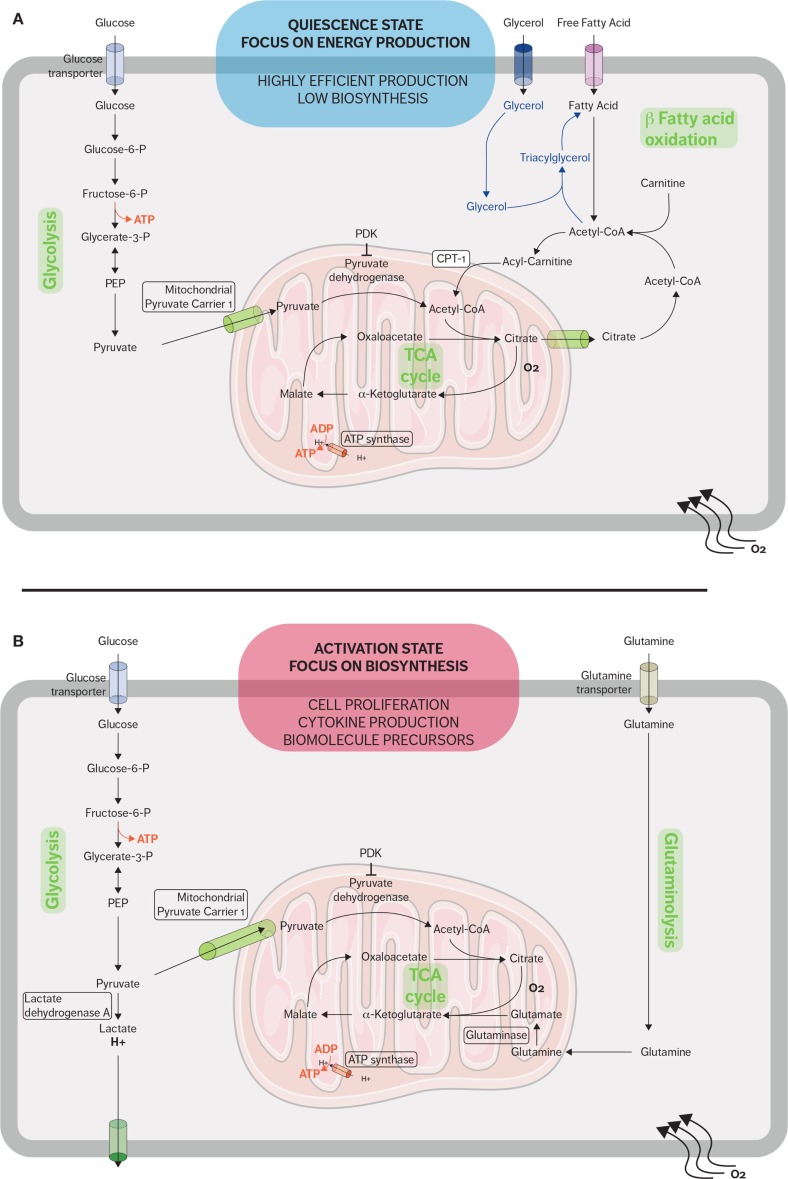
**Metabolic pathways used by CD8 T cells in quiescent and active states**. **(A)** When in a quiescent state, naive CD8 T cells fulfill their energy needs mainly through aerobic glycolysis and mitochondrial respiration. Fatty acid oxidation plays an important role during the restriction phase when effector CD8 cells differentiate into memory CD8 cells and the immune system returns to a quiescent state. Memory T cells synthesized fatty triacylglycerols to supply fatty acids for fatty acid oxidation. **(B)** When stimulated, effector CD8 T cells rapidly upregulate glycolysis and glutaminolysis because the activated cells require many of the metabolic intermediates for macromolecule biosynthesis. The metabolic adaptations sustain the rapid expansion of effector CD8 T cells and support the secretion of cytokines and cytotoxic molecules.

In addition to mitochondrial glucose oxidation, resting T cells generate energy through the β-oxidation of fatty acids and especially quiescent memory subsets (Figure 1A). Fatty acid oxidation (FAO) is integral to the development of memory T cells, and when FAO is impeded, the memory CD8 T-cell population is heavily affected (35, 36). Pearce et al. showed that mice which lack tumor necrosis receptor-associated factor 6 (TRAF6) cannot upregulate FAO, and while these mice were able to mount a normal effector CD8 response, they were unable to form a CD8 memory population after bacterial infection (37). Additionally, TRAF6-deficient CD8 cells could not properly activate AMP-activated kinase (AMPK); however, metformin, an AMPK activator, was able to rescue FAO in the TRAF6-deficient CD8 cells and promote the development of the memory subset (37). Interestingly, the fatty acids used are not acquired from their external environment. Instead, FAO is supported in memory T cells by the synthesis of triacylglycerols (TAGs) from externally acquired glucose. IL-7 has been shown to upregulate the expression of glycerol channel aquaporin 9 (AQP9) and increased TAG synthesis in antigen-experienced CD8 T cells. The resulting TAGs are hydrolyzed by lysosomal acid lipase into free fatty acids for FAO (36, 38). The generation of potent CD8 memory T cells is also dependent on mTOR-related signaling since rapamycin, a mTOR inhibitor, promotes memory T-cell differentiation (39, 40). Rapamycin treatment during the CD8 expansion phase has been shown to increase the resulting quantity of CD8 memory cells; furthermore, rapamycin given during the contraction phase results in memory CD8 cells that are highly functional and can mount a better memory response (39, 41, 42).

### Activation of CD8 T Cells Leads to Metabolic Reprogramming

Proliferation and potent effector functions are an energetically demanding process that requires a metabolic adaptation in order to fulfill the needs of T cells. Upon activation, CD8 T cells reprogram their metabolism from OXPHOS to aerobic glycolysis and glutaminolysis (Figure 1B) (5). This glycolytic shift supports the rapid proliferation of activated lymphocytes not only in terms of energy requirement but also in terms of metabolic intermediates (43). These metabolic intermediates allow the biosynthesis of macromolecules essential for the massive cell growth and proliferation required as effector CD8 cells expand during the immune response. For example, these intermediates generated from the glycolytic pathway are used to generate nucleotides, amino acids, and lipids, which in turn will be used to synthesize nucleic acids, lipids, and proteins needed in order to duplicate the cellular biomass (8, 43).

**Figure 2:**
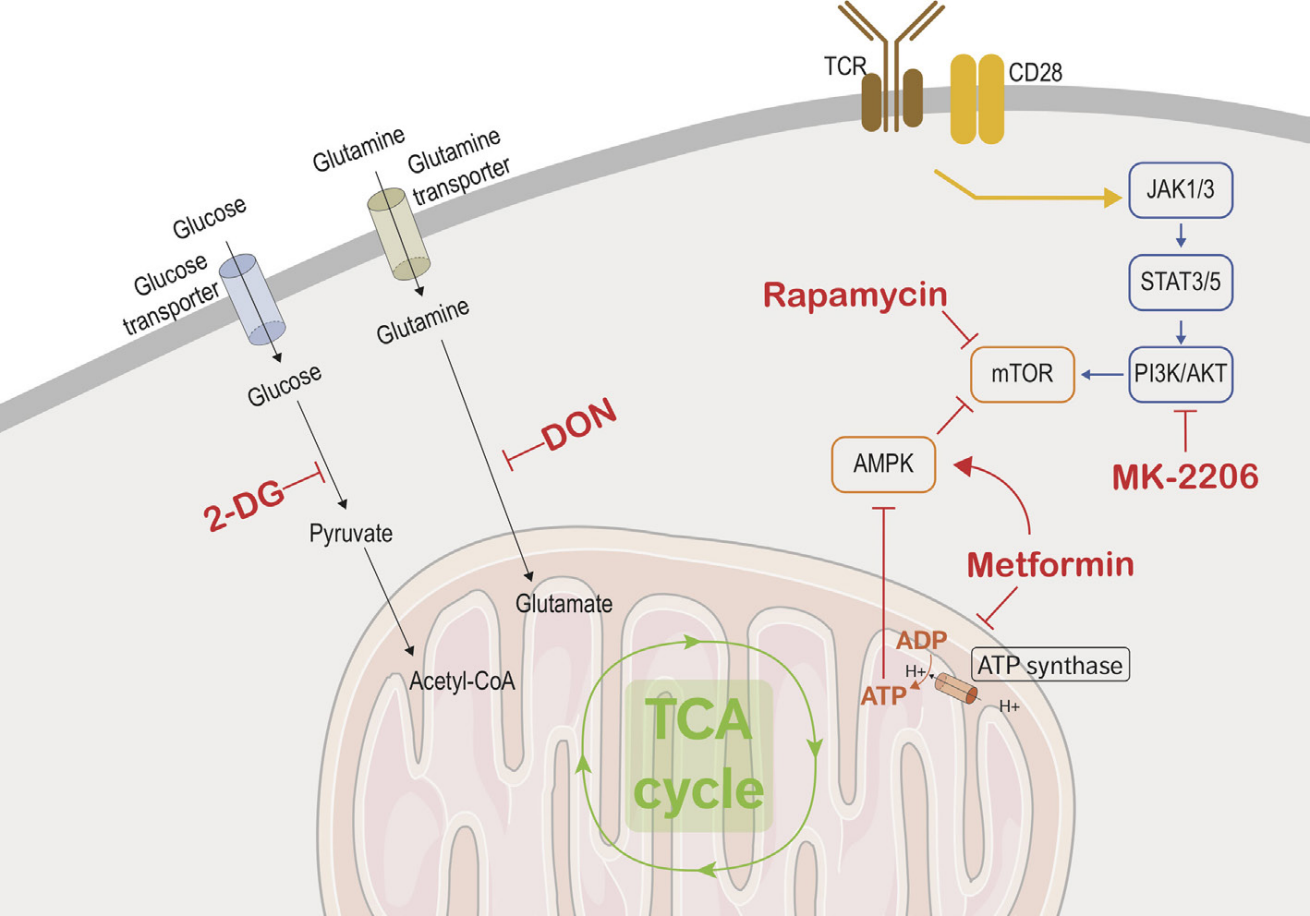
**Immunometabolic targets**. This figure depicts the different metabolic and transcriptional pathways involved in the immunometabolic functions of CD8 T cells. Black arrows are used to show the transport of glucose and glutamine into the cells where they are metabolized. The yellow arrow represents the combined signaling of the TCR and CD28 which activates CD8 effector cells and triggers the metabolic adaptations in the effector cells. The blue arrows show the transcriptional used by activated CD8 T cells to upregulate mTOR, which controls cellular metabolism and growth. Red arrows show the promotion or suppression of therapeutic molecules on the various immunometabolic pathways.

T-cell activation requires two signals: one from the T-cell receptor (TCR) and the other from costimulatory receptors. TCR signaling alone is not sufficient for inducing major changes to the metabolic programming (44). The increased glucose metabolism seen in activated lymphocytes is due to CD28 costimulation signaling, which in turn activates the PI3K/AKT pathway (Figure 2) (44–46), leading to an increase of nutriment uptake, cell-surface expression, and function of glucose transporter. However, it is becoming apparent that there are specific metabolic adaptations dependent on the T-cell subset. Gubser et al. showed that the effector memory CD8 T cells are able to switch to glycolysis more rapidly as compared to naive CD8 T cells (47). Furthermore, effector memory CD8 T cells are able to sustain this higher glycolytic rate. Glycolysis has also been implicated in posttranscriptional control of cytokine secretion in activated lymphocytes. Glyceraldehyde-3-phosphate dehydrogenase (GAPDH) is an enzyme involved in the glycolytic process and has been shown to act as an mRNA-binding protein. Chang et al. demonstrated that interferon-γ (IFNγ) secretion was suppressed in activated lymphocytes which were cultured in galactose and thus could not utilize the glycolytic pathway. The depression in the IFNγ secretion is due to the binding of GAPDH to IFNγ transcripts. Therefore, glycolysis in lymphocytes has a secondary role as a regulator of GAPDH binding of IFNγ (48). The drastic diversion of energy generation from OXPHOS to aerobic glycolysis induced by T-cell activation may be oversimplified as a higher mitochondrial mass of memory CD8 T cells compared to naive CD8 T cells has been reported and linked to a higher enhancement of both OXPHOS and glycolysis in memory CD8 T cells (49).

In addition to increased glycolytic activity, activated T cells also upregulate glutamine metabolism (glutaminolysis). Glutamine, a common amino acid found in human plasma, can serve as an alternative source of energy. Activated lymphocytes require glutamine for cell proliferation and cytokine secretion, and CD28 costimulation enhances glutamine uptake and increases glutamine transporter expression (50, 51). Several metabolic TCA cycle intermediates, such as citrate, α-ketoglutarate, and oxaloacetate, are crucial for proliferation as precursor for lipid and amino acids synthesis. Glutamine undergoes anaplerotic reaction to produce oxaloacetate and α-ketoglutarate metabolism through glutamate. Glutaminolysis also replenishes NADPH pool, which is also needed by proliferating lymphocytes to support lipid and nucleotide biosynthesis as well as maintaining the redox balance (51, 52).

## Metabolic Targets for Immunomodulating CD8 T Cells

There are two possible approaches to manipulating the metabolic programming of CD8 cells as a means of immunosuppression for transplantation. The first would be to target metabolic pathways that are involved in cytokine production and cytotoxic activity of effector and effector memory cells in order to shut down CD8 T cells’ ability to cause damage to allografts (Figure 2). The other method would be to target metabolic pathways to enhance and promote the proliferation of CD8 Tregs, which will in turn cause a suppressive effect on the CD8 effector population, thus prolonging allograft half-life and possibly favoring tolerance induction.

### Shutting Down the Warburg Effect in Effector CD8 T Cells

As previously discussed, glycolysis and glutaminolysis are two key metabolic pathways that are imperative for proper CD8 effector function. Finding methods to interrupt these pathways in allograft infiltrating effector cells would be a mean stopping allograft rejection. 2-Deoxy-d-glucose (2-DG) is a glucose analog that inhibits glycolysis by blocking hexokinase function. Many studies showed the potent effect of 2-DG in inhibiting the cytotoxic function of effector CD8 cells (47, 53–55). In a similar fashion, blocking glutaminolysis with a glutamine antagonist such as 6-diazo-5-oxo-l-norleucine (DON) inhibits lymphocyte proliferation (56). Additionally, blocking glucose and glutamine transporters disrupt lymphocyte activation and affect memory differentiation (57–60).

Targeting major bioenergetic pathways seems at first glance a rather dangerous means to control an immune response and major side effects such as a massive toxicity to normal tissue is expected. Animal models and ongoing therapeutic use of metabolic interferences therapies prove that such strategy is safe and feasible. For instance, leflunomide and its active metabolite teriflunomide prevent *de novo* biosynthesis of pyrimidine and efficiently inhibit the development of EAE (61). In multiple sclerosis patients, teriflunomide, compared to placebo, significantly reduces relapse rates, disability progression (at the higher dose), and MRI evidence of disease activity (62). Interestingly, whereas a link with metabolism has not been established, leflunomide or analogs have also been shown to be effective in prolonging graft survival and even to induce tolerance in a model of heart allograft transplantation (63). Metabolic interferences have been mainly studied in the cancer field. Given the similarity of metabolic adaptation between cancer cells and activated T cells, immunometabolic regulation of CD8 T cells could be used as a means to manipulate the CD8 T-cell immune function for effective immunosuppression. However, the doses required to eradicate all malignant cells and those required to control auto-or alloreactive T cells might be radically different. Cancer therapy aims to eradicate all cancer cells, whereas transplant therapy aims to control alloreactive T cells. Given their effector nature, alloreactive T cells are likely to be characterized by a higher use of glycolysis as compared to quiescent T cells. The selective targeting of glycolysis processes will thus focus preferentially alloreactive and not quiescent (naive or memory) T cells.

### Targeting Transcriptional Regulators of Immunometabolism

Since metabolic adaptation is required to support T-cell activation and function, nutrient availability or limitation will affect these processes. A recent publication has shown that adenosine monophosphate-activated protein kinase (AMPK) couples T-cell function to nutrient availability (64). AMPK is a serine-threonine kinase that is sensitive to energy levels and is activated during cellular stress. By sensing the AMP/ATP ratio, AMPK senses energy deficiency and favors pathways leading to ATP production while inhibiting ATP-consuming pathway. It increases catabolic processes and inhibits anabolic processes to increase ATP production when activated. AMPK upregulates fatty acid β-oxidation by promoting the transfer of long-chain fatty acids into the mitochondria via carnitine palmitoyltransferase 1 (CPT1) (5). Metformin, a drug commonly used in diabetes treatment, blocks mitochondrial complex I, which has the downstream effect of promoting AMPK activity. Interestingly, metformin fosters memory CD8 T-cell differentiation in mice (37). In agreement with these results, it has been shown that autoreactive T cells can be efficiently controlled by the coadministration of 2-DG and metformin in a mouse model of systemic lupus erythematosus (SLE) (65). However, as meformin also inhibits OXPHOS, *in vitro* or *in vivo* administration is likely to have a broader target than solely the memory compartment.

The PI3K/AKT pathway is another key pathway that integrates immune stimulation and nutrient uptake (9). Blocking PI3K/AKT pathway would therefore be another way to suppress the effector function of CD8 cells. The core kinases of this pathway are AKT, AMPK, and mTOR. An intimate positive and negative cross-regulation of these protein kinases has been shown and this topic has been covered in-depth in several publications (5, 6, 9). AKT pathway is optimally activated by the coligation of TCR and CD28 and leads to the increase of glycolysis, via an increase of glucose uptake and the enhancement of rate-limiting glycolytic enzymes hexokinase and phosphofructokinase (66). Preventing activation of this pathway could be accomplished by inhibiting the costimulation signaling provided by CD28 or by directly inhibiting AKT activity. FR104, an anti-CD28 antagonist antibody, has been shown to prevent lymphocyte activation and proliferation in a murine model (67). Furthermore, this drug has been shown to be effective in reducing allograft rejection in both murine and non-human primate models of transplantation (68, 69). Another possible strategy would be to target AKT directly through the use of one of the AKT inhibitors currently in development (70). For example, AKT inhibitor MK-2206 treatment in a murine model increased the differentiation of naive CD8 cells into central memory CD8 cells and diminished terminal differentiation in the CD8 population (71). Additionally, AKT can be modulated upstream by inhibiting focal adhesion kinase (FAK), a well-established regulator of the PI3K/AKT pathway. Inhibiting FAK in Ewing sarcoma cells results in downregulation of both AKT and mTOR and impaired cell growth and colony formation (72, 73). Donor lymphocyte infusions following allogeneic stem cell transplantation are performed to enhance the graft-versus-tumor (GVT) effect, and minor histocompatibility antigen (MiHA)-specific CD8 T cells play an important role in this GVT response. It has been hypothesized that adoptive MiHA-specific CD8 T-cell transfer would lead to a more efficacious GVT response while also minimized graft-versus-host disease (GVHD), a harmful effect which is also observed in allogeneic stem cell transplant patients. AKT signal inhibition during *ex vivo* priming of naive precursor cells resulted in the generation of stem cell-like MiHA-specific CD8 T cells. Additionally, these cells have a superior proliferation capacity and antitumor effects in a murine model (74). While clinical research on these developmental AKT inhibitors has been mainly focused on the field of oncology and allogeneic stem cell transplantation, there could be possible use for them in the solid organ transplantation field.

### Boosting Treg Populations with Metabolic Programming

In the field of CD4 Tregs, there has been a lot of progress in finding viable ways to use them in clinical practices. Several clinical trials are currently in progress to evaluate the safety and efficacy of adoptive transfer of CD4 Tregs in allograft transplant recipients (75). However, studies concerning CD8 Tregs are at more exploratory stages.

Rapamycin is an antifungal macrolide that is produced by bacteria discovered on Easter Island which targets and inhibits mTOR and is already used as a part of immunosuppressive regimens in transplantation. mTOR is a serine/threonine kinase that regulates cell survival, growth, and energy metabolism. mTOR can form two distinct complexes, mTOR complex 1 (mTORC1), which is rapamycin sensitive, and mTOR complex 2 (mTORC2), which is rapamycin insensitive and much less studied compared to mTORC1 (41, 76–79). mTORC1 is the master regulator of cell growth and metabolism and can be activated through either PI3K or AKT signaling. Upon activation, mTORC1 promotes ribosome biogenesis and increases protein translation and synthesis (6, 78, 79). mTORC1 also promotes lipid biosynthesis and regulates mitochondrial metabolism and biogenesis through sterol regulatory element-binding proteins (SREBP) which are involved in positively regulating lipid homeostasis (80, 81). Rapamycin targets and inhibits mTOR activity, thus interfering with G1 phase cell cycle activity and inhibits interleukin-2 (IL-2)-driven proliferation in T lymphocytes. This leads to a potent immunosuppressive effect. Consequently, mTOR inhibitors have been adopted into use as a part of immunosuppressive regimens in solid organ transplantation (82, 83). In addition to the immunosuppressive effects of rapamycin, it has also been shown to induce the preferential growth of CD4^+^CD25^+^FOXP3^+^Tregs *in vitro* (84). Evidence is emerging that rapamycin treatment *in vitro* can also induce CD8^+^CD28^−^Tregs in murine models (85), indicating that adoptive transfer strategies to induce allograft tolerance that are currently under investigation for CD4 Tregs could potentially be adjusted for CD8 Tregs.

Another immunosuppressive drug that has been shown to be able to induce preferential expansion of CD8 Tregs *in vitro* is CTLA-4-Ig belatacept, a recently approved drug for transplant immunosuppression treatment (86). A CTLA-4 antagonist would affect the metabolic function of effector CD8 cells by blocking CD28 costimulation signaling, which has the downstream effects of impeding AKT activation and downregulating glucose and glutamine uptake. Additionally, Barbon et al. showed that by first alloanergizing human PBMCs with belatacept and then allostimulating the cells caused an increase in the frequency of CD8^+^CD28^−^ T cells. Furthermore, repeated rounds of allorestimulation after alloanergization would continue to expand the CD8^+^CD28^−^ Tregs, which were able to suppress the proliferation of CD4 T cells in a dose-dependent manner (86). Additionally, Barbon et al. reported on three hematopoietic stem cell transplant recipients with a marked increase in blood circulating CD8^+^CD28^−^ T cells between days 20 and 40 post-transplantation (86). While these findings are still preliminary, it is promising evidence that CD8 Tregs could play a future role in controlling allograft rejection.

## Conclusion

When CD8 T cells go from a quiescent to an activated state, there are also major alterations to their metabolic programming. Upregulation of glycolysis and glutaminolysis has been shown to be important not only to fulfill the energy requirements of the activated effector CD8 cells but also to provide the biosynthetic materials needed for this rapidly expanding population. Research into the immunometabolics of CD8 cells not only expands our understanding about CD8 T cells but also provides new pharmaceutical targets that could help to reduce the negative effect of these cells in an allograft immune response.

## Conflict of Interest Statement

The authors declare that the research was conducted in the absence of any commercial or financial relationships that could be construed as a potential conflict of interest.
